# Composite Pheochromocytoma-Paraganglioma With Ganglioneuroma: A Dual-Center Clinical Experience

**DOI:** 10.1210/jendso/bvaf036

**Published:** 2025-02-26

**Authors:** Shubham Agarwal, Hussam Alkaissi, Karel Pacak, Jorge Esteban Mosquera Izurieta, Alan P Dackiw, Sarah C Oltmann, Fiemu Nwariaku, Liwei Jia, Mary Grace Roden, Oksana Hamidi

**Affiliations:** Division of Endocrinology and Metabolism, University of Texas Southwestern Medical Center, Dallas, TX 75390, USA; National Institute of Diabetes and Digestive and Kidney Diseases, National Institutes of Health, Bethesda, MD 20892, USA; Section on Medical Neuroendocrinology, Eunice Kennedy Shriver National Institute of Child Health and Human Development, National Institutes of Health, Bethesda, MD 20892, USA; Division of Endocrinology and Metabolism, University of Texas Southwestern Medical Center, Dallas, TX 75390, USA; Department of Surgery, University of Texas Southwestern Medical Center, Dallas, TX 75390, USA; Department of Surgery, University of Texas Southwestern Medical Center, Dallas, TX 75390, USA; Department of Surgery, University of Utah, Salt Lake City, UT 84112, USA; Department of Pathology, University of Texas Southwestern Medical Center, Dallas, TX 75390, USA; Harold C. Simmons Comprehensive Cancer Center, University of Texas Southwestern Medical Center, Dallas, TX 75390, USA; Division of Endocrinology and Metabolism, University of Texas Southwestern Medical Center, Dallas, TX 75390, USA

**Keywords:** neuroendocrine neoplasia, adrenal tumor, catecholamines

## Abstract

**Context:**

Cells derived from neural crest populate several organs. A particular precursor cell, sympathogonia, gives rise to pheochromoblasts and neuroblasts. Due to common origin, tumors originating from pheochromoblasts, such as pheochromocytoma (PHEO) and paraganglioma (PGL), may rarely coexist with ganglioneuroma (GN).

**Objective:**

We evaluated clinical, biochemical, and radiological characteristics of patients with composite PHEO/PGL and GN (PPGL-GN) and compared them to patients with PHEO.

**Methods:**

In this retrospective, dual-center, observational, case-control study, we identified patients with PPGL-GN. Similarly, we identified a control group of patients with PHEO who underwent laparoscopic adrenalectomy. All diagnoses were confirmed on histology. Descriptive statistics were used to summarize demographic and clinical data.

**Results:**

We identified 19 consecutive patients with PPGL-GN and 86 patients with PHEO. Patients with PPGL-GN, compared to those with PHEO, were younger (aged 46.0 vs 50.8 years; *P* = .03), had higher rate of underlying genetic disorders (47.4% vs 23.2%; *P* = .03), and had fewer functioning tumors (89.5% vs 100%; *P* = .002). There was no difference in the median radiological tumor size or the precontrast computed tomography density. Disease recurrence (at another site) was noted in 15.8% of PPGL-GN patients who had a median follow up of 14.6 months, as opposed to no disease recurrence in patients with PHEO. There was no documented recurrence at the tumor bed and no metastasis in both groups.

**Conclusion:**

Patients with PPGL-GN were younger and had a higher occurrence of underlying genetic disorders compared to PHEO. However, PPGL-GN was radiologically indistinguishable from PHEO. The higher observed disease recurrence of PPGL-GN reinforces vigilant postoperative follow-up.

During embryonic development, the neural crest cells migrate and populate several organs, giving rise to specialized cells, including the precursor cell, sympathogonia, which in turn gives rise to catecholamine-secreting chromaffin cells in the adrenal medulla and sympathetic paraganglia [1]. Pheochromocytoma (PHEO) arises from the chromaffin cells of the adrenal medulla and produces excessive amounts of catecholamines. Paraganglioma (PGL) arises from the extra-adrenal paraganglia. Another derivative of neural crest cells are the Schwann cells that are widely present in peripheral nerves but can be associated with adrenal tumors.

Up to 30% to 40% of adult patients with PHEOs and PGLs (PPGLs), and 80% of pediatric patients, have known susceptibility germline mutations such as multiple endocrine neoplasia (MEN) 2A, MEN 2B, neurofibromatosis 1 (NF1), von Hippel-Lindau disease (VHL), as well as hereditary PPGL syndrome due to mutations of genes encoding for succinate dehydrogenase (SDH) subunits [2-4].

Ganglioneuroma (GN) is usually a benign, differentiated tumor of the autonomic ganglion cells, which is also derived from neural crest cells [5]. GNs typically arise in the retroperitoneal space, and approximately 30% are located in the adrenal glands [6]. Adrenal GNs are usually nonfunctioning and sporadic but can occasionally have a syndromic presentation [6] and ectopic hormone production [7].

Due to the common origin of both tumor cells, PHEO can have components of other neural crest–derived cells (GN, ganglioneuroblastoma, or schwannoma) (Fig. 1). These tumors are classified as composite PHEO [8]. Composite PHEOs are rare, and there is considerable uncertainty about their clinical presentation and radiological features.

**Figure 1.**
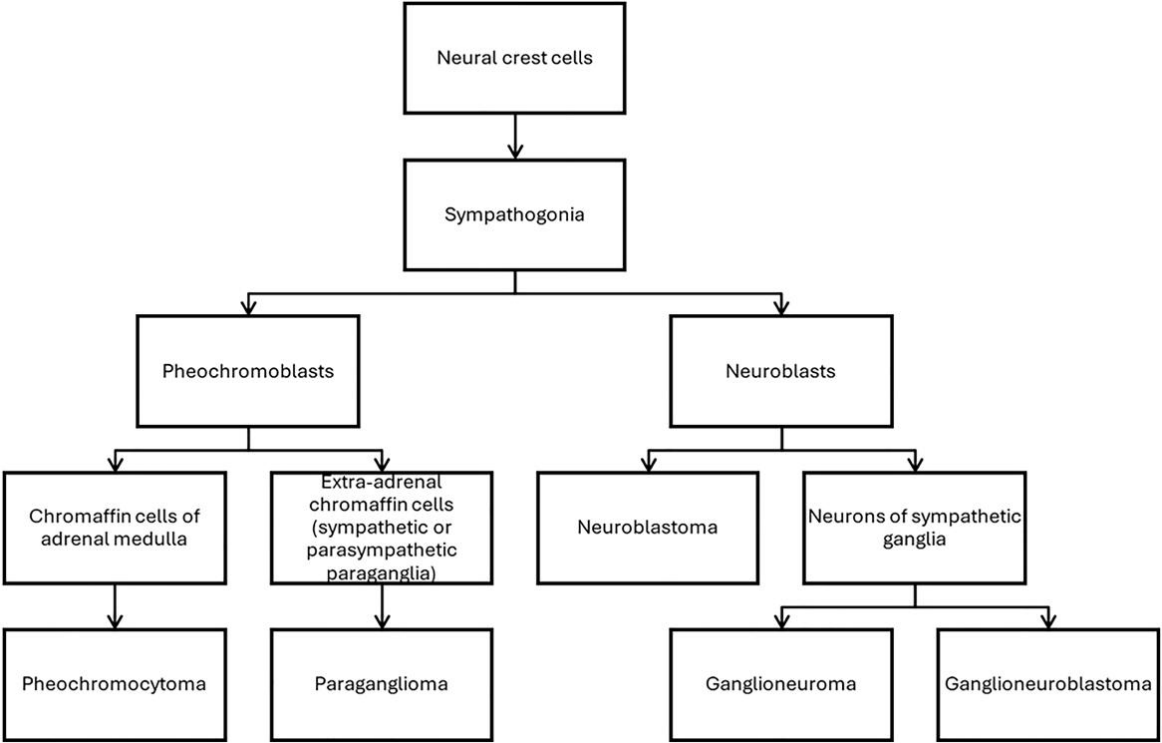
Developmental lineage of neural crest cells. Neural crest cells differentiate into sympathogonia, which further develop into pheochromoblasts or neuroblasts. Pheochromoblasts give rise to chromaffin cells, which can form pheochromocytoma when located in the adrenal medulla or paraganglioma when these cells are extra-adrenal. Neuroblasts can mature into neurons of sympathetic ganglia or form neuroblastomas if they undergo neoplastic transformation. In some cases, neuroblastomas can mature into ganglioneuromas.

We conducted a retrospective, longitudinal, dual-center case-control study with the aim of describing clinical, biochemical, and radiological features of patients with composite PPGL with GN (PPGL-GN) and contrasting those to patients with PHEOs.

## Materials and Methods

### Study Design and Population

This retrospective, longitudinal, case-control study comprised consecutive patients seen at 2 academic tertiary care medical centers—University of Texas Southwestern Medical Center (UTSW) in Dallas, Texas, USA, and National Institutes of Health Clinical Center (NIH) in Bethesda, Maryland, USA. This study was approved by the respective institutional review board of both centers. Identification of eligible patients was done by reviewing pathology and surgery databases and by searching the electronic health record (EHR) system for the encounter and problem list diagnosis codes. A search of the EHR at UTSW revealed 584 cases of PPGL and GN. All identified patients' EHRs were then reviewed to confirm histologic diagnosis of PPGL-GN. At UTSW, we identified 10 cases of composite PPGL-GN between January 1, 2006, and December 31, 2023, and an additional 9 cases were identified by searching the NIH database. The control group comprised 86 patients with histologically confirmed PHEO who underwent minimally invasive adrenalectomy at UTSW during the study period. Demographic and clinical data were extracted from the EHR. The data extraction included demographic characteristics (age, sex, patient-reported race and ethnicity, family history), diagnosis-related information (histological tumor diagnosis, date of initial diagnosis, mode of tumor discovery, recurrence, metastasis, specialty encounter history), clinical characteristics (laterality, biochemical laboratory values, imaging data), and genetic testing results. Disease recurrence was defined as the appearance of a structural tumor at any site and/or biochemical abnormalities after complete primary tumor resection and normalization of previously elevated biochemical markers. All tumors were defined as functioning when urine or plasma fractionated or total metanephrines were elevated above the upper limit of respective reference ranges. The reference intervals for plasma and urine metanephrines were established at Mayo Clinic [9]. The tumors were considered nonfunctioning when the levels of plasma and/or urine metanephrines/catecholamines remained within the reference ranges. Tumors hypersecreting primarily epinephrine/metanephrine or norepinephrine/normetanephrine were termed *adrenergic* and *noradrenergic*, respectively.

Patient consent was waived with no identifying information present in the publication of the manuscript.

### Data Analysis

Descriptive statistics were used to provide a summary of all data. All continuous variables were summarized as median (minimum to maximum range), while categorical variables were summarized as absolute and relative frequencies (percentages). Associations between variables were assessed using the *t* test and analysis of variance for continuous variables and chi-square test for categorical variables. *P* value less than .05 was considered statistically significant. All statistical analyses were conducted using JMP version Pro 18 (SAS Institute) and Microsoft Excel for Microsoft 365 version 2408 (Microsoft Corporation).

## Results

### Baseline Characteristics

We identified 19 consecutive patients with PPGL-GN. Of these, 15 (79%) patients had PHEO-GN, 3 (16%) had PGL-GN, and 1 (5%) patient had PHEO-ganglioneuroblastoma. The control group comprised 86 patients with PHEOs. Table 1 lists baseline characteristics of the studied population.

**Table 1.** Baseline demographics and tumor characteristics displayed by the nature of tumor (PPGL-GN and PHEO)

Variable	Composite PPGL-GN (n = 19)	PHEO (n = 86)	*P*
Median age at diagnosis, y (SD)	46 (19.0)	50.8 (14.9)	.03
Female, n (%)	12 (63.0%)	47 (54.7%)	.49
Race			.86
White, n (%)	13 (68.4%)	63 (73.3%)	
African American, n (%)	2 (10.5%)	9 (10.5%)	
Asian, n (%)	3 (15.8%)	6 (6.9%)	
Unknown/Undisclosed, n (%)	1 (5.3%)	8 (9.3%)	
Ethnicity			.07
Hispanic, n (%)	5 (26.3%)	9 (10.5%)	
Non-Hispanic, n (%)	14 (73.7%)	77 (89.5%)	
Presence of family history of PPGL, n (%)	2 (10.5%)	9 (10.5%)	.99
Germline mutation and somatic pathogenic variants/genetic syndrome, n (%)	9 (47.4%)	20 (23.2%)	.03
Mode of discovery			.21
Incidental, n (%)	8 (42.1%)	44 (51.1%)	
Symptom driven, n (%)	7 (36.8%)	33 (38.4%)	
Other cancer surveillance, n (%)	2 (10.5%)	4 (4.7%)	
Germline mutation driven, n (%)	1 (5.2%)	5 (5.8%)	
Biochemically functioning, n (%)	17 (89.5%)	86 (100%)	.002
Adrenergic, n (%)	13 (76.5%)	63 (73.3%)	.77
Noradrenergic, n (%)	4 (23.5%)	23 (26.7%)	
Biochemical test elevation*^a^*, median (IQR)			
Plasma metanephrine	4.2 (2.0-7.0)	4.3 (2.4-13.6)	.46
24-h urinary metanephrine	4.3 (1.3-7.5)	5.0 (2.4-14.8)	.48
Plasma normetanephrine	7.2 (1.8-11.3)	4.8 (2.5-9.4)	.73
24-h urinary normetanephrine	2.4 (2.3-3.9)	3.3 (2.3-8.3)	.34
Median tumor size, cm (IQR)	3.8 (2.5-4.7)	3.7 (2.7-5.2)	.73
Median precontrast CT density, HU (IQR)	37 (20-40.2)	40 (27.7-44)	.48

Abbreviations: CT, computed tomography; GN, ganglioneuroma; HU, Hounsfield units; IQR, interquartile range; PHEO, pheochromocytoma; PPGL, pheochromocytoma/paraganglioma.

^a^Data expressed as fold increase in reference to upper limits of normal.

The median age at diagnosis of PPGL-GN was 46.0 years and 50.8 years in the PHEO group (*P* = .03). Both groups had a female sex preponderance: 63.0% in PPGL-GN and 54.7% in PHEO. No clinical differences were noted between race, ethnicity, or the presence of family history of PPGL. More patients with PPGL-GN (47.4%) had an underlying genetic disorder compared to PHEO (23.2%) (*P* = .03), with MEN 2A being the most common disorder in both groups (Table 2). Other underlying genetic syndromes in patients with PPGL-GN included MEN 4, MEN 2A VHL, NF1, Pacak-Zhuang syndrome, Chuvash polycythemia, and mutations in genes encoding SDH subunits B, C, and D.

**Table 2.** Germline and somatic pathogenic variants in patients with composite pheochromocytoma/paraganglioma-ganglioneuroma

Genetic syndrome or germline/somatic pathogenic variant
MEN 2A, *RET*, germline, p.C634Y (c.1901G > A)
MEN4, *CDKN1B*, germline, p.M52R (c.155T > G)
NF1, *NF1*, germline, c.499_502del (p.Cys167GInfs*10)
*SDHB*, germline, exon 3 deletion
*SDHC*, germline, exon 1, c.20 + 13insTG
*SDHD*, germline, exon 3 splice-site mutation, c.170-1G > T
VHL, *VHL*, germline, p. R200W
VHL, *VHL*, germline, p.T124I
*EPAS1*, somatic, p.A530V

Abbreviations: EPAS-1, endothelial PAS domain protein 1; MEN, multiple endocrine neoplasia; NF1, neurofibromatosis type 1; SDH, succinate dehydrogenase; VHL, von Hippel-Lindau.

### Clinical, Biochemical, and Radiological Features

Most cases were detected either incidentally (42.1% in PPGL-GN vs 51.1% in PHEO) or due to catecholamine-related symptoms (36.8% in PPGL-GN vs 38.4% in PHEO). Although not statistically significant, we observed a trend of higher PPGL-GN discovery rate during cancer surveillance (10.5%), compared to 4.7% of PHEOs. Germline mutation–driven case detection rates were similar in both groups (5.2% in PPGL-GN vs 5.8% in PHEO).

The median tumor size was similar in both groups: 3.8 cm (IQR, 2.5-4.7 cm) in PPGL-GN and 3.7 cm (IQR, 2.7-5.2 cm) in PHEO. The median precontrast radiological density of the tumor was 37 Hounsfield units (HU) (IQR, 20-40.2 HU) for PPGL-GN and 40 HU (IQR, 27.7-44 HU) for PHEO.

**Figure 2.**
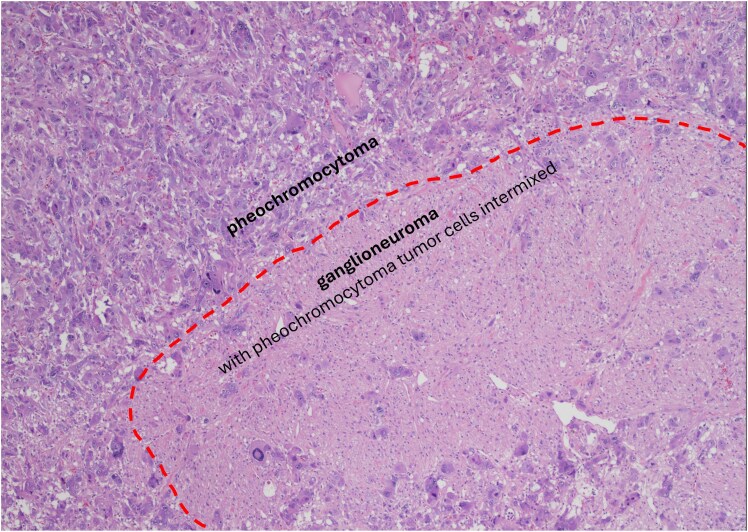
A histological section of composite pheochromocytoma-ganglioneuroma. The tumor is composed of two components (marked by a broken line): pheochromocytoma characterized by tumor cells with abundant fine, granular red-purple cytoplasm, and ganglioneuroma (focally intermixed with pheochromocytoma, as shown at the lower right corner) characterized by admixture of ganglion cells and Schwann cells embedded in the Schwannian stroma (×40 magnification).

All PHEOs and most PPGL-GNs (89.5%) were biochemically functioning, with similar distribution of adrenergic and noradrenergic biochemical phenotypes (see Table 1). The median fold elevation in PPGL-GN vs PHEO group was 4.2 (IQR, 2.0-7.0) vs 4.3 (IQR, 2.4-13.6) for plasma metanephrine, 4.3 (IQR, 1.3-7.5) vs 5.0 (IQR, 2.4-14.8) for 24-hour urine metanephrine, 7.2 (IQR, 1.8-11.3) vs 4.8 (IQR, 2.5-9.4) for plasma normetanephrine, and 2.4 (IQR, 2.3-3.9) vs 3.3 (IQR, 2.3-8.3) for 24-hour urine normetanephrine, respectively. None of the included patients in either group had metastatic disease. The median duration of follow-up from initial diagnosis was 18.5 months (IQR, 5.4-118.7 months) and 9.8 months (IQR, 4.0-50.5 months) for patients with PPGL-GN and PHEO, respectively. Similarly, the median postoperative follow-up was 14.6 months (IQR, 3.0-67.8 months) and 1.1 months (IQR, 0.5-15.8 months) for PPGL-GN and PHEO, respectively. Disease recurrence was noted in 15.8% PPGL-GN patients, and no disease recurrence was noted in patients with PHEO. There was no tumor recurrence in the primary tumor bed. A histological section of a PPGL-GN is presented in Fig. 2.

## Discussion

In this retrospective, longitudinal, dual-center, case-control study of the largest reported composite PPGL-GN cohort, we found that patients with PPGL-GN presented at a younger age and had a higher association with underlying genetic syndrome than patients with PHEO. PPGL-GN and PHEO tumors both had a female sex predilection. Despite PPGL-GN tumors having another component, there were no differences in the mode of presentation when compared to PHEO, with most patients either being diagnosed incidentally or due to catecholamine-related symptoms. Our study showed that PPGL-GNs were more commonly biochemically functioning tumors, with most having an adrenergic biochemical phenotype. The higher normetanephrine elevation in PPGL-GN is possibly due to the PPGL component preferentially producing normetanephrine as seen in certain PPGL with underlying genetic syndromes [10] but was not statistically significant in our study; otherwise, biochemical profiles were similar. No major differences were observed in the radiological tumor dimensions or precontrast HU density between the two types of tumors.

Our findings complement the results from another large series of patients with PPGL-GN, which showed that PPGL-GN are commonly brought to medical attention either incidentally or due to patient-reported symptoms of catecholamine excess [11]. Another study that summarized 94 published PPGL-GN cases found similar modes of discovery [12]. In addition, the authors reported a female sex preponderance, similar to our study.

Consistent with our findings, PPGL-GNs are commonly biochemically functioning tumors with a female sex predilection [11]. While the prior case series reported that the median age at diagnosis of PPGL-GN tumors was higher (52 years) than our findings (46 years), the median age of diagnosis of PPGL-GN in the present study was lower than that of patients diagnosed with PHEO, which suggests that PPGL-GNs tend to develop at a younger age [11]. In a series of previously published PPGL-GN cases, the median age at diagnosis was 48 years, which was consistent with our findings [12]. The earlier age of diagnosis in the present study is likely a result of a higher incidental discovery of tumors, compared to the case series where the mode of detection commonly was symptom driven. Another possible explanation for the earlier age at diagnosis in patients with PPGL-GN is a higher prevalence of the underlying genetic syndrome.

No major differences in precontrast radiodensity between the two types of tumors were noted in the prior study, as well as our study [11]. Of note, prior studies reported larger median tumor size of PPGL-GN cases [11, 12]. In contrast, our study showed a lower median tumor size both of PPGL-GN and PHEO tumors, compared to the prior studies, and this is likely due to advancements in imaging techniques over time, difference in the mode of discovery between both studies, and a higher awareness of PPGL in the health-care community over recent years. Higher median tumor size in prior studies likely reflects the symptom-driven mode of discovery as opposed to more tumors being discovered incidentally.

We found a higher rate of underlying genetic disorder in patients with PPGL-GN. MEN 2A was the most common mutation, while NF1 was more common in the meta-analysis [12]. The cohort of patients with PHEO in our study was similar to the largest published cohort of PHEO cases with regard to mode of discovery, median age at diagnosis, female sex predilection, tumor size at diagnosis, precontrast radiodensity, and occurrence of underlying genetic diseases [13].

Our study adds to the existing literature on PPGL-GN and PHEO by highlighting baseline characteristics and clinical outcomes of patients with these rare tumors. While this is the largest study of PPGL-GN cohorts from distinct medical centers, it has a number of limitations. The retrospective design of the study limits the findings to documentation accuracy in the medical records, and confounders could be present. Another challenge with a retrospective design is that of missing data in EHR (eg, imaging data, tumor proliferation indices, immunohistochemical staining), and we undertook careful chart review to overcome this challenge and were able to obtain additional data. Lastly, patients with PHEO had a short postoperative duration of follow-up since most patients transitioned their postoperative follow-up outside UTSW, which could potentially underestimate the risk of PHEO recurrence. Despite the limitations, the results in this study have the potential to influence clinical practice.

In summary, PPGL-GNs were biochemically functioning tumors that occurred at a younger age than PHEO but appeared radiographically indistinguishable from PHEO. PPGL-GN had a stronger association with an underlying genetic disorder than previously reported. This calls for increased attention to perform genetic testing on such individuals. The high disease recurrence of PPGL-GN warrants vigilant postoperative follow-up.

## Data Availability

Data contained were gathered under an IRB protocol with institutional patient data; further inquiries can be directed to the corresponding author. The data underlying this article will be shared on reasonable request to the corresponding author.

## Abbreviations

EHR: electronic health record
GN: ganglioneuroma
HU: Hounsfield units
IQR: interquartile range
MEN: multiple endocrine neoplasia
NIH: National Institutes of Health Clinical Center
NF1: neurofibromatosis 1
PGL: paraganglioma
PHEO: pheochromocytoma
PPGL: pheochromocytoma/paraganglioma
SDH: succinate dehydrogenase
UTSW: University of Texas Southwestern Medical Center
VHL: von Hippel-Lindau disease
